# Population genomics of East Asian ethnic groups

**DOI:** 10.1186/s41065-020-00162-w

**Published:** 2020-12-08

**Authors:** Ziqing Pan, Shuhua Xu

**Affiliations:** 1 Key Laboratory of Computational Biology, CAS-MPG Partner Institute for Computational Biology, Shanghai Institute of Nutrition and Health, University of Chinese Academy of Sciences, Chinese Academy of Sciences, Shanghai, 200031 China; 2 School of Life Science and Technology, ShanghaiTech Universit, Shanghai, 201210 China; 3 Center for Excellence in Animal Evolution and Genetics, Chinese Academy of Sciences, Kunming, 650223 China; 4 Collaborative Innovation Center of Genetics and Development, School of Life Sciences, Fudan University, Shanghai, 200438 China

**Keywords:** Population genetics, Whole-genome sequence data, East Asian, Evolutionary forces

## Abstract

East Asia constitutes one-fifth of the global population and exhibits substantial genetic diversity. However, genetic investigations on populations in this region have been largely under-represented compared with European populations. Nonetheless, the last decade has seen considerable efforts and progress in genome-wide genotyping and whole-genome sequencing of the East-Asian ethnic groups. Here, we review the recent studies in terms of ancestral origin, population relationship, genetic differentiation, and admixture of major East- Asian groups, such as the Chinese, Korean, and Japanese populations. We mainly focus on insights from the whole-genome sequence data and also include the recent progress based on mitochondrial DNA (mtDNA) and Y chromosome data. We further discuss the evolutionary forces driving genetic diversity in East-Asian populations, and provide our perspectives for future directions on population genetics studies, particularly on underrepresented indigenous groups in East Asia.

## Background

In the past two decades, novel methods were developed and facilitated in the generation of large genomic data that researchers have used to improve our understanding of the population genetic architecture and evolutionary history of humans [1]. However, most genetic studies are based on populations of European ancestry. Non-European populations such as East Asian (EA) and African are underrepresented. The lack of ethnic diversity in human genetic studies impedes our understanding of the panorama of both the ancient human migration and the present-day human population diversity [2].

**Fig. 1:**
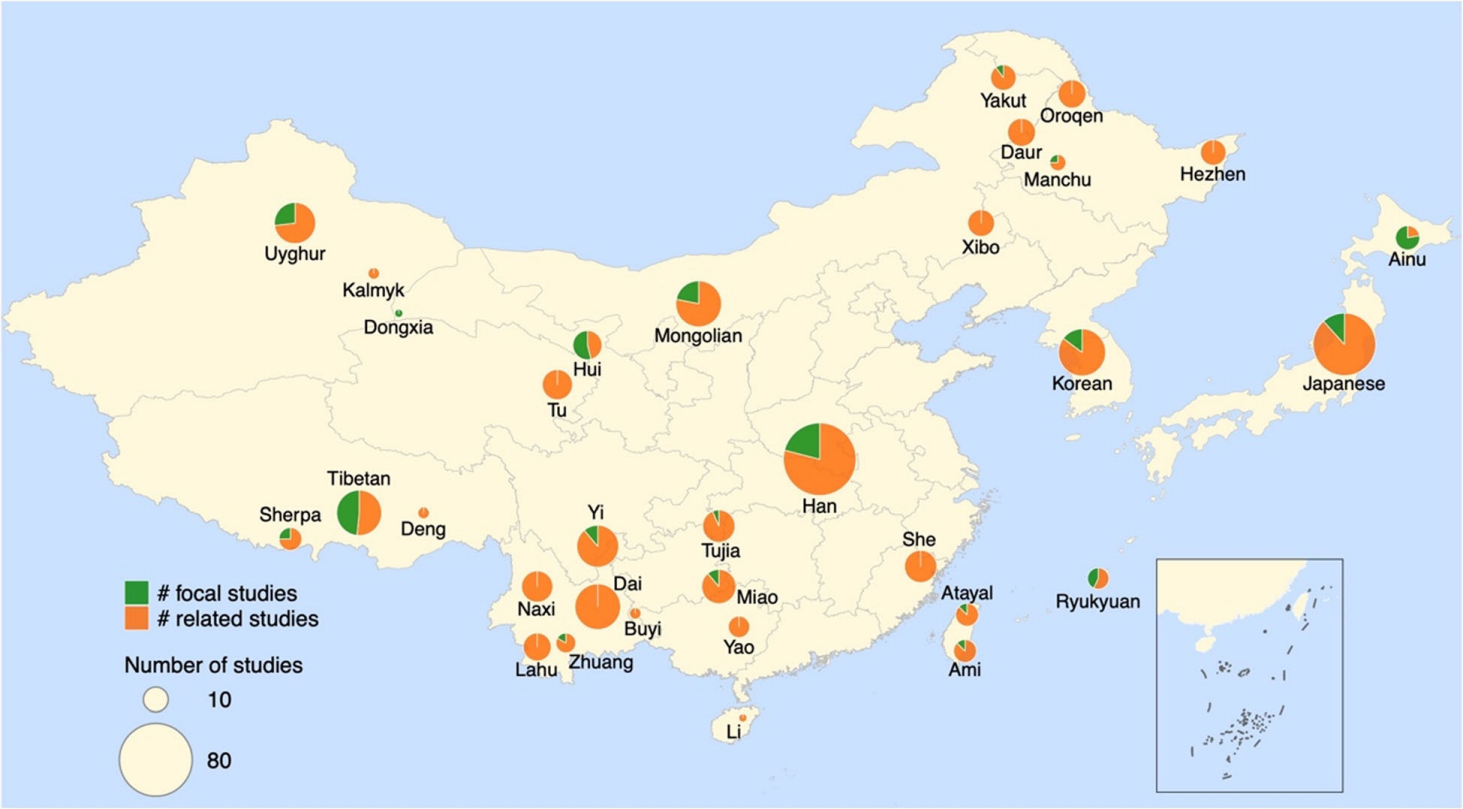
A graphical summary of population genetic studies on EA populations. The summary is based on population genetics studies on EA populations from 2009 to 2020 retrieved from online database PubMed (https://pubmed.ncbi.nlm.nih.gov/) (search string query: East Asian and Population genetics). We have retrieved a total of 1960 papers, of which 91 are directly related to the population genetics among East Asian groups and are counted in the summary. Focal studies stand for studies that specifically focus on the target populations while related studies are those that include the target populations. Geographical locations of populations studied are roughly annotated

East Asians represent about 38% of the Asian population and 22% of the global population. Their population occupied a unique geography, located on the crossroads connecting the Americas and Pacific Islands, which plays a pivotal role in human evolutionary history. The whole-genome population genetic studies on East Asians can be traced back to 2009, when the HUGO Pan-Asian SNP Consortium (HUGO-PanAsia) reported the first cohort of large-scale genome data on Asians [3]. Prior to that, population genetics studies in East Asian mainly relied on sparse markers on mtDNA and the Y chromosome [4–8]. These studies have proposed the historical models of EA population formation, origins, subsequent population migration and division, and impact of social practices on current populations. During the subsequent genome-wide data era, with higher-coverage data and improved analytic methods, some previous findings were supported and validated, while others were rejected or confirmed. We summarize the results of recent population genetics studies on EA populations since 2009 (Fig. 1), although some populations were covered but not specifically studied by some studies that aimed to investigate global population diversity.

Here, we review the recent progress in population genomics of East Asia, concentrating on three typical populations, namely, Chinese, Japanese and Korean. We describe differences in the genetic architectures of these populations, explore the mechanisms that maintain the genetic diversity among EA populations, and decipher the evolutionary scheme of East Asians. This review improves our understanding of East Asian population genetic structure and more importantly, emphasizes the need for additional genetic studies on the under-represented ethnic groups.

## Population genetic studies on east Asian ethnic groups

According to geographical distribution and ethnicity, East Asians can be roughly divided into three major groups: Chinese, Korean and Japanese. Although these three populations share many physical similarities, genomic studies show that the genetic makeup of the these populations differ from each other [9, 10]. Therefore, in this section, we provide a general overview of the genetic ancestry, subpopulations, admixture, and migration history of each group.

### Japanese populations

According to the studies based on mtDNA, Y chromosomes and genome-wide sequence data, the historical periods of Japan follow a dual structural model that consists of three periods [11–13]. The first Paleolithic period dates back to 14,500 years ago (ya), followed by the Jomon period spanning from 14,500 to 2300 ya, and the third Yayoi period from 2300 to 1700 ya. The periods also correlate with Japanese ancestries. Jomon are the ancient hunter-gather population from Southeast Asia, while Yayoi are later agriculture-migration-related immigrants from Northeast Asia.

These ancestries have different genetic contributions to various present-day Japanese sub-populations. The Japanese population is currently divided into three separate groups: mainland Japanese (also called Hondo), Ryukyuan, and Ainu [12]. Mainland Japanese is the major population located in the central continent of Japan. It is regarded as the descendant of Jomon and Yayoi, with a higher genetic contribution from Yayoi than the other two populations. Ryukyuan is located in Okinawa in the southern islands of Japan, while Ainu is an indigenous population in Hokkaido and southern part of Sakhalin islands, north of Japanese archipelago. The Ryukyuan population, which can also be divided into three subpopulations (Okinawa in the north, Miyako in the middle, Yaeyama in the southwest) based on both geographic and genetic distances, received major genetic components from Korean and Jomon [12, 14]. Genetic drift possibly accounts for the genetic differences among the Ryukyuan subgroups rather than admixture. The Ainus are genetically closest to Jomon and are regarded as the offspring of Jomon, with later disparate gene flows from other East Asian populations, including mainland Japanese, resulting in genetic heterogeneity in Ainu [15]. It’s also genetically closer to Ryukyuan than mainland Japanese, possibly due to the high-proportion of shared Jomon-ancestry. Previous studies based on Y chromosome data suggest a genetic relationship between Tibetan and Ainu [16]. However, this correlation was not confirmed by whole-genome data analyses [17]. Recent findings indicate that Ainu is more closely related to low-altitude East Asians than high-altitude East Asians.

### Korean populations

The Korean Peninsula is located to the north of China and its northeast region is bound by Russia. The Korean population has been thought to be highly homogeneous with few admixtures in its history [10, 18, 19]. Previous studies based on mtDNA and Y chromosome show that the Korean ethnic group received a large proportion of genetic components from Northeast Asia and a small proportion from Southeast Asia, which suggests a south-to-north migration route to Korea [20]. Moreover, reanalysis of Y-chromosome data showed the southern genetic contribution in females is less than that in males, indicating a male-biased migration pattern, which is possibly associated with the spread of rice agriculture [21]. Similarly, a study based on genome-wide SNP data also supports the male-biased south-to-north migration [22]. A recent study employing more whole-genome data of both present-day and ancient populations recaptured the two major genetic components from East Siberia and Southeast Asia [18]. The study also unveiled the origin of the admixed genetic components, which suggests an initial admixture between Tianyuan and Devil’s gate ancestries throughout East Asia and East Siberia until the Neolithic era, followed by a more recent admixture with ancient Southern Chinese populations in the Bronze Age and ultimately a migration to Korea. The recent admixture with Southeast Asians, including Chinese and Cambodians, contributes to the genetic makeup of present-day Korean subpopulations.

### Chinese populations

Chinese is the largest population in Eastern Asia, consisting of 56 officially identified ethnic groups, with Han Chinese as the major ethnic group. The minorities are distributed throughout the country; for instance, Tibetans and Sherpas in the Qinghai-Tibet Plateau, Uyghurs in western China, Dais in southern China, Mongolians in central and northern China. Extensive research studies have been made to uncover the genetic structure and evolutionary history of Chinese populations. One remarkable genetic pattern is the distinction between northeastern and southeastern Chinese groups, based on phylogeographic studies of mtDNA variations and non-recombined Y chromosome (NRY) haplogroups [4, 6, 7]. These studies have supported the north-south division with greater diversity in southern EA, indicating a north-to-south migration route although without direct evidence. Strong support is provided by the HUGO-PanAsia study [3], which shows a highly significant correlation between haplotype diversity and latitude, and confirmed by a maximum-likelihood analysis. This study offers compelling evidence for a south-to-north direction of early EA migration although other movements from north to south remain esoteric.

Except for the north-to-south cline, later genome-wide studies on select minority population such as the Uyghurs in Xinjiang also show a west-to-east cline, which is associated with an admixture between EAs and Europeans [23]. Xinjiang is located in western China and is bound by eight countries, namely, Mongolia, Russia, Kazakhstan, Kyrgyzstan, Tajikistan, Afghanistan, Pakistan, and India. It serves as the key place of the ancient Silk Road, which links the East and the West of Eurasia. Its unique geography may contribute to the high diversity of the Xinjiang population. Uyghurs, the most typical indigenous population in Xinjiang, are the key ethnic group for inferring the history of recent genetic exchange between eastern and western Eurasian. Studies on mtDNA and the Y chromosome confirmed Uyghurs were derived from both eastern and western Eurasian people [24, 25], which are the descendants of the most ancient Turkic tribes with mixed Caucasian and East-Asian ancestries. A recent genome-wide scale study further shed light on the ancestry and origin of the Uyghurs [26]. It separates the two components into four specific origins, which consist of European (24.9-36.6%), South Asian (12.0-19.9%), Siberian (15.2–16.8%), and East Asian (28.8–46.5%). Based on the different compositions of the genetic make-up, the Uyghur population could be divided into two sub-populations, namely, the northeast and southwest. This population structure is associated with longitude instead of latitude, which may result from a joint effect of the barrier of the Tianshan Mountain and gene flow from Eastern and Western neighboring Eurasian populations. Researchers also estimated the admixture time and proposed a two-wave admixture model. First, West Europeans and Southern Asians admixed as the Western component (WE-SA) in 5000–3750 ya, whereas the Eastern Asians and Siberians met in the East and formed as the Eastern ancestries (EA-SIB) in 5500–5000 ya. Subsequently, the mixed WE-SA and EA-SIB joined together as the founder of Uyghur’s gene pool. The West-East contact possibly occurred twice, one in around 3500 ya and the next in 750 ya.

Another essential minority for inferring human population history is the Tibetan ethnic group. Settled in the highest plateau with an average elevation of > 4500 m worldwide are the Tibetan high-landers constituting a distinct population in China. It is estimated that 90% of the Tibetan genome was inherited from modern humans and about 6% originated from mixed archaic hominoids, including Neanderthals (~ 1%) and Denisovans (0.4%) [27]. Some archaic segments that remained in the Tibetan genome are crucial for high-altitude adaptation [28]. Tibetans are genetically closest to East Asians among the global populations, and closer to other plateau ethnics, including Tu, Yi and Naxi than lowlanders such as Han Chinese. Current methods assumed that the Tibetan and Han shared EA components, taking up > 80% of their genome, and the divergent time was about 15,000–9000 ya. The admixture model for Tibetan evolution could be summarized as a two-wave admixture model that is similar to that of Uyghur’s evolution. The first ancient wave could be dated back to before the last glacial maximum (LGM; 26,500-19,000 ya), when some archaic hunter-gathers like Denisovans, Neanderthals, ancient Siberians met on the plateau and formed an archaic group known as SUNDer, as a result of admixtures among Siberians, an Unknown archaic group, Neanderthals, and Denisovans. The later admixture occurred in post-LGM between SUNDer’s descendants and lowland modern human groups represented by Han Chinese, which contribute to the majority of the ancestry of the present-day Tibetans.

Mongolians are another important ethnic group that has been used in inferring EA population history. Located in central and northern China, southern Russia, and other neighboring countries, Mongolians play a pivotal role in shaping the culture and genetic makeup of modern Eurasia along with the Mongolian Empire expansion in the thirteenth century based on mtDNA and Y chromosome data [29, 30]. Genome-wide data further uncover gene flow from Europeans to Mongolians, noting that Mongolian have ~ 10% European ancestry [31]. Contemporary Mongolian populations can be divided into six distinct tribes, namely, the Abaga, Khalkha, Oirat, Buryat, Sonid and Horchin, based on geographical distribution. Oirat has the highest genetic diversity among the tribes, possibly due to the forming of a relatively small and isolated population during its history. Buryat is the most differentiated group from other EA populations, while Horchin is the least. It has been estimated that the divergence time between Mongolian and other EA populations is around 13,000 to 7000 ya except for Horchin in around 4500 ya [32]. The divergence time between Oirat and Horchin is around 7000–5500 ya, while Buryat separated from the remaining Mongolian groups 4000–2000 ya. Another Mongolian group are the Deedu Mongolians, who migrated from the Mongolian steppes to the Qinghai-Tibetan Plateau and shared adaptive genes with Tibetan such as *EPAS1*, *PKLR,* and *CYP2E1* [33]. This group comprises more Tibetan components (about 52%) and less Mongolian components (about 44%) than the other Mongolian group like Buryat (75% Mongolian component and 25% EA component). Studies on the Mongolian population could provide valuable insights into the admixture between East and West Asian and genetic adaptation to extreme environments.

Most of the genetic variations among populations of non-European ancestry are enigmatic, which may affect both disease prediction and treatment efficacy. Moreover, the unique genetic mosaic of minority populations, like the SUNDer in Tibetan’s genome, is the distinguished material that has been to comprehend the function and phenotypic effect of selected variations, bypassing genome-wide association studies (GWAS) or traditional laboratory research. Therefore, in the future, it is essential that more data could be collected to better understanding the genetic structure of human populations.

## Relationship and origin of EA groups

In the previous section, although we discussed the genetic pictures of three EA groups separately, no geographic region can be regarded as isolated in human population history, especially in terms of migration and admixture that form the basal genetic materials of present-day EA [6]. Therefore, we further discuss the origin and relationship of the EA populations.

Based on mtDNA, NRY, whole-genome sequencing data and recent ancient DNA data, the common ancestor in EA is characterized as two layers of ancestry: pre-Neolithic hunter-gatherers as the first layer and northern East Asians since Early Neolithic as the second layer [6, 18, 34]. The two layers of ancestry admixture contribute to the basal genetic architecture of present-day EAs, while subsequent regional migration and admixture introduce additional genetic variations among the EA populations. The most pronounced distinction is the north-to-south cline of genetic patterns among EA, i.e., the haplotype diversity is strongly correlated with latitude. This pattern is confirmed by studies on mtDNA, Y chromosome, and autosomal variations [3, 6, 8], and a recent study further proved that the south-to-north division could be traced back to Early Neolithic, more than 7500 ya [34]. Intuitively, it raises the question of whether the early migration route occurred in the north-to-south direction or the opposite? The findings of research investigations on mtDNA and NRY haplogroups have remained controversial. However, the HUGO-PanAsia study has provided compelling evidence for a south-to-north direction of EA population spread, including a higher haplotype diversity in the south and higher proportion of Southeast Asian haplotype that shared among EAs, coupled with a maximum-likelihood tree and phylogenetic reconstruction using group private haplotypes analysis, which all pointed to south-to-north migration events and unified the field [3, 6].

These ancestries mixed in EA compose the embryonic gene pool of present EA continent populations, while later migration and human expansion events have built neighboring Japanese and the Korean populations. Based on genetic differentiation (*F*_ST_) and effective population size (*N*_e_) inferred from the modern genome data, researchers estimate the divergence time between present-day Han Chinese and Japanese to be ~ 3600-3000 ya, and the divergence time between Han Chinese and Korean as ~ 1200 ya while Japanese and Korean separated ~ 1400 ya [10]. Subsequent gene flows among the three populations after divergence can be determined by F-statistics and D-statistics. For example, based on a F_3_ test, gene flow occurred from Chinese and Japanese to Korean and between Han and Japanese populations. These admixtures partly attenuate the population differentiation and homogenize the groups.

Except for admixture among local EA populations, gene flow from other regions outside EA, such as western Eurasian and South Asia, also made crucial contributions to the EA populations. A research based on D-statistics showed west-to-east cline genetic pattern among Han Chinese, indicating continuous admixing source from West Eurasian in the northwestern provinces of China [23]. Similarly, another D-statistics based study unearthed that the Ainus share ancestry with northeast Siberians [17]. Taken together, these findings facilitate our understanding of present-day population genetic structures and relationships.

## Evolutionary forces driving genetic diversity of EA populations

Mutation, genetic admixture, genetic drift and natural selection are noted as major driving forces that contribute to the genetic diversity among populations. Because new mutations rarely play a major role in the evolution of genetic diversity in human populations, which is particularly true in closely related EA populations, we mainly focused on the latter three mechanisms and illustrate their roles in forming the genetic diversity of present-day EAs.

### Genetic admixture

As earlier discussed, ancient migration and admixture shaped the basal gene pool of EAs, and subsequent admixture events among the groups are expected to reduce the genetic differentiation between the three ethnic groups. Conversely, regional gene flow from surrounding populations and different proportions of migration can accentuate the population diversity. For instance, although with a common ancestor, pairwise F_ST_ between Han Chinese and Japanese and that between Han Chinese and Korean are both greater than that between north and south Hans [10]. Other population analyses, such as K-mean, STRUCTURE, and principal component analysis (PCA), can also distinguish Han Chinese, Korean, and Japanese as three distinct groups [3, 18]. The population structure is mostly related to the different proportions of gene flow sources to these populations. It has been reported that the major source of gene flow to Han Chinese was from southern ethnic groups, the major source of gene flow to Japanese was from southern islands, while the major source to Koreans were from both mainland and islands [10]. Moreover, Koreans received more gene flow from the Chinese while the Japanese show a closer genetic relationship with Koreans. Therefore, those admixture events contribute to further genetic diversity of the three groups.

Another case is the Sherpa, an indigenous population in the Qinghai-Tibet Plateau. The Sherpa were once regarded as one of the ancestral sources of the Tibetans [35]. However, recent analyses revealed that Sherpas received higher levels of South Asian ancestry while Tibetans showed a higher proportion of EA and Central Asian ancestries, which rejected the hypothesis and suggested a demographic model with multiple waves of migration and admixture [36].

Except for continental populations, gene flow also contributes to the genetic diversity of archipelagic populations. As we have discussed above, both PCA and genetic clustering analysis show that Ainu people are genetically heterogeneous and even form a few distinct clusters. The long-term admixture between Ainu and mainland Japanese accounts for its genetic diversity [11, 15, 17].

In conclusion, these studies demonstrate that gene flow and admixture from surrounding groups contribute to the genetic diversity of populations. Moreover, because admixture can rapidly change the gene pool in one generation and introduce novel genetic materials for adaptation, it serves as the principle driving force that causes and retains genetic diversity and is also a crucial element for inferring human evolutionary genetics.

### Genetic drift

After ancient migration and admixture shaped the basal genetic pool for EA, genetic drift plays an indispensable role in generating genetic diversity among regional subpopulations, especially in the archipelago. Population structure-based studies, including ADMIXTURE and PCA, showed genetic diversity among Ryukyuan sub-populations, while the D-statistics did not significantly depart from zero, which suggests that genetic drift plays a predominant role in shaping the genetic structure among Ryukyu landers [14]. It has been reported that the EA groups have a similar but not identical demographical history. For instance, although they all underwent strong population expansion about 20,000 ya, Han Chinese has greater N_e_ than the Japanese and the Koreans. Whereas, the Korean population expanded faster than the Japanese in the last thousands of years [10]. These uneven rates of population expansion may strengthen the genetic drift in archipelagos and peninsulas, which is expected to increase population genetic diversity.

### Natural selection

After the population settles down in a particular place with a certain environment, natural selection might play an important role in driving population differentiation, especially when mutation is rare and genetic drift is weak in large populations. Previous studies have shown that some genes are associated with adaptation in EA which also results in genetic differentiation between EA and other non-EA populations. These EA-specific genes include *EDAR* (ectodysplasin A receptor), *FADS* (fatty acid desaturase), *OCA2* and *ADH* (Alcohol dehydrogenase) gene [22, 37–39]. *EDAR* has a variety of pleiotropic effects, including sweat gland density, incisor shoveling, and mammary gland ductal branching. A nonsynonymous V370A mutation in the *EDAR* gene has been reported to have elevated derived allele frequency in North and East Asians and associate with “East Asian phenotypes. Further studies have confirmed the *EDAR* gene harbors a strong selective sweep signal that is associated with an increase in the number of active eccrine glands during the LGM. *FADS* genes encode rate-limiting enzymes for the biosynthesis of long-chain fatty acids and underwent positive selection in multiple populations, including EA [22, 23]. A recent study on the high-altitude environment of the Beringia proposed an alternative hypothesis that the selective context for EDARV370A acted on the allele’s effect of increasing ductal branching in the mammary gland instead of sweat gland density in EA populations and this intertwined with selection on the *FADS* gene [40]. Under the condition of extremely low UV radiation during LGM, people in Arctic Beringia may experience vitamin D deficiency, which leads to reduced absorption of calcium, and compromised immunological and adipose tissue function. However, the selected *FADS* genes help modulate the relative proportion of long-chain polyunsaturated fatty acids during breast milk synthesis under low-vitamin D condition. In contrast, vitamin D deficiency is relevant to an increase in mammary ductal branching during the hormone-induced stages of breast development, which is established via the NF-κB signaling pathway and that is activated by *EDAR*. In conclusion, selection for polymorphisms in the *FADS* gene cluster and for EDARV370A may result from the intertwined advantage in transmitting nutrients from mother to infant through breast milk in the low UV environment. The *ADH* gene has three subtypes, *ADH1A*, *ADH1B,* and *ADH1C*. An ADH1B Arg47His variant increases the alcohol metabolism rates and is predominant in EAs but rare in Europeans and Africans. The positive selection signal and culture-related selective forces on this gene have been proposed. Further population studies elucidated an east-to-west cline (98.5% in southeastern China, 60–70% in western China) in the allele frequency distribution in EAs with a relatively low frequency in Sherpa and Tibetan (10–20%) [38]. Molecular dating suggests the emergence time of the allele was about 10,000–7000 ya and the spread of ADH1B Arg47His was possibly correlated to rice domestication in China, resulting in the disparity in allele frequency among populations.

Except for the shared adaptive genes, selection also contributes to the genetic differentiation among closely related populations. We have reviewed the genetic diversity between Sherpas and Tibetans resulted from admixture and migration; however, natural selection also imparted an effect. Although Sherpas and Tibetans shared some plateau adaptive genes including *EPAS1, EGLN1*, and *TMEM247*, some genes were specifically underlying natural selection in Sherpa, such as *ALDH3A1, ANGPT1*, and *OXR1* [36, 41]. These genes are related to adaptation to hypoxia and high levels of ultraviolet radiation environment shared between Sherpas and Tibetans. Nevertheless, the difference in allele frequency between these two highland groups has proven that selection contributes to population genetic differences.

Overall, selection and adaptation are complex processes that yield different consequences in the population genome, which may increase or decrease the genetic diversity.

## Perspectives

The increasing availability of data, especially whole-genome data, largely facilitates our understanding of the genetic mechanisms and evolutionary history of EAs (Table 1). Although we can now decipher a sketch of EA evolutionary history, the definitive genetic relationship and evolutionary processes among the subpopulations in EA remain unclear. Findings have been reported that soft selective sweeps on standing variants with higher fixation probability and faster adaptation rate comprised about 92.2% of all human sweep signatures [52–54]. Most novel and rare variants can only be detected in regional populations. For example, utilizing the rare and low-frequency variants associated with height in the Japanese, researchers have reported 573 height-associated variants and two novel height-associated genes [55]. The rarer variants tend to have height raising effects, suggesting negative selection on height-increasing alleles in the Japanese, which is contrary to the findings in European, showing that rarer variants have height-decreasing effects. This finding calls for additional researches in subpopulations to better fathom the evolutionary process or even envisage the future evolutionary direction, we are obliged to place more effort on related studies.

**Table 1:** A summary of recent studies on EA populations

Population	Ethnic Group	Data	Reference
Japanese	Mainland Japanese, Ainu and Ryukyuan	MtDNA and Y chromosome	[16]
		Microarray genotyping	[10, 11, 14, 15, 17, 39]
		Whole-genome sequencing	[13, 42]
Korean		MtDNA and Y chromosome	[21]
		Genotyping	[10, 19, 43]
		Whole-genome sequencing	[18, 44]
Chinese	Han Chinese	MtDNA and Y chromosome	[4, 7, 45]
		Microarray genotyping	[3, 10]
		Whole-genome sequencing	[23, 46–48]
	Tibetan and Sherpa	Microarray genotyping	[35, 36, 41, 49]
		Whole-genome sequencing	[27, 36, 50]
		Third-generation sequencing	[51]
	Uyghur	MtDNA and Y chromosome	[24, 25]
		Microarray genotyping	[26]
	Mongolian	MtDNA and Y chromosome	[29, 30]
		Microarray genotyping	[31]
		Whole-genome sequencing	[32, 33]

Another gap lies on studies of the structural variations (SVs) of human populations. The history of study of structural variants can be traced back to the early twentieth century. Current studies have mostly focused on variations such as SNPs and microsatellites in the scope [56]. Recent advances in high-throughput next-generation sequencing and the third-generation sequencing open a new window to study the role of SVs in human adaptation and evolution. For instance, using a high-quality Tibetan genome (ZF1), researchers revealed a 163-bp intronic deletion in the *MKL1* gene that is associated with lower systolic pulmonary arterial pressure, which is a crucial adaptive trait in Tibetans [51]. As many more high-quality genome assemblies are available, we expect that a more comprehensive picture of SVs at both individual and population levels will be constructed.

The biological meaning of human genome sequence remains at its infancy. The functions and phenotypic effects of the majority of our genome are unknown. There are three typical approaches to understand the biological meaning of our genome sequence: i) medical studies start from a certain phenotype (or disease) and aim to identify the corresponding genotype; ii) model-organism-based experimental studies starting from a gene by knocking in/out some sequences to observe its molecular function or phenotypic effects; and iii) evolutionary studies evaluate functional importance of many variants in parallel across the genome by estimating their conservation or adaptive potential.

The medical approach relies on phenotypes observed, and results are usually inconsistent among different studies due to poor phenotyping; experimental approaches are expensive, inefficient and can only be done in non-human organisms. Evolutionary analysis of diverse populations is equivalent to doing direct knock-in/out studies (actually occurred in nature) in human genomes, thus is an economic and efficient way to understand biological meaning of our genome sequence. For instance, by whole-genome sequencing 1055 healthy Korean individuals, the Korean Variant Archive database has reported 293,049 variants, of which 88,047 (30%) variants are novel compared with the dbSNP database [57]. Functional assessment of the non-synonymous variants supported the purifying selection signal in Koreans and a list of rare functional variants have been reported to be associated with increased cancer susceptibility, which could inspire subsequent biomedical research.

Moreover, comprehensive studies on population genetics also facilitate our understanding of biological meaning of our genome data, especially in the GWAS. First of all, population stratification is a significant confounding factor in GWAS. Researchers have uncovered that the genetic differentiation among the Han Chinese, although very small (F_ST_ = 0.0002–0.0009), is sufficient to induce an inflated false-positive rate with a moderate sample size [22]. The problem of missing heritability also hinders our interpretation of GWAS results, which are based on the assumption of common disease/common variants hypothesis [58]. To better explain the genotype-phenotype relationships, we need more lower-frequency variants that may contribute to an extensive fraction of the heritability of common diseases.

There is thus a need for additional studies that focus on under-investigated indigenous populations, such as people living in tropical forest and highland in EA and Southeast Asia, whose genomes harbor an enormous number of variants that might not have been observed in earlier population studies, especially those of European ancestry.

## Conclusion

Previous studies have taken advantage of population genetic data, especially whole-genome sequence data to illuminate the evolutionary history of EA populations. In this review, we summarize recent researches and focus on novel evolutionary insights on three EA groups, namely, Chinese, Japanese and Korean, and illuminate how a wide range of evolutionary forces including migration, admixture, genetic drift and natural selection form the populations while driving population diversity. Finally, we anticipate additional investigations on under-researched indigenous minor populations as well as fine maps of high-quality sequence data to resolve the genetic structure of human genetics.

## Data Availability

Not applicable.

## Abbreviations

mtDNA: Mitochondrial DNA
EA: East Asian
HUGO-PanAsia: the HUGO Pan-Asian SNP Consortium
Ya: Years ago
NRY: Non-recombined Y chromosome
LGM: Last Glacial Maximum (26,500-19,000 ya)
SUNDer: An admixed group of ancestries derived from ancient Siberians, Unknown archaic groups, Neanderthals, and Denisovans
GWAS: Genome-wide association studies
N_e_: Effective population size
PCA: Principal component analysis
SV: Structural variation

## References

[1] Bustamante CD, De La Vega FM, Burchard EG (2011). Genomics for the world. Nature.

[2] Sirugo G, Williams SM, Tishkoff SA (2019). The missing diversity in human genetic studies. Cell.

[3] Abdulla MA, Ahmed I, Assawamakin A, Bhak J, Brahmachari SK, Calacal GC, Chaurasia A, Chen C-H, Chen J, Chen Y-T, Chu J, la Paz EMC C-d, MCA DU, Delfin FC, Edo J, Fuchareon S, Ghang H, Gojobori T, Han J, Ho S-F, Hoh BP, Huang W, Inoko H, Jha P, Jinam TA, Jin L, Jung J, Kangwanpong D, Kampuansai J, Kennedy GC, Khurana P, Kim H-L, Kim K, Kim S, Kim W-Y, Kimm K, Kimura R, Koike T, Kulawonganunchai S, Kumar V, Lai PS, Lee J-Y, Lee S, Liu ET, Majumder PP, Mandapati KK, Marzuki S, Mitchell W, Mukerji M, Naritomi K, Ngamphiw C, Niikawa N, Nishida N, Oh B, Oh S, Ohashi J, Oka A, Ong R, Padilla CD, Palittapongarnpim P, Perdigon HB, Phipps ME, Png E, Sakaki Y, Salvador JM, Sandraling Y, Scaria V, Seielstad M, Sidek MR, Sinha A, Srikummool M, Sudoyo H, Sugano S, Suryadi H, Suzuki Y, Tabbada KA, Tan A, Tokunaga K, Tongsima S, Villamor LP, Wang E, Wang Y, Wang H, Wu J-Y, Xiao H, Xu S, Yang JO, Shugart YY, Yoo H-S, Yuan W, Zhao G, Zilfalil BA, The HUGO Pan-Asian SNP Consortium (2009). Science.

[4] Li H, Cai X, Winograd-Cort ER, Wen B, Cheng X, Qin Z, Liu W, Liu Y, Pan S, Qian J, Tan C-C, Jin L (2007). Mitochondrial DNA diversity and population differentiation in southern East Asia. Am J Phys Anthropol.

[5] Rosenberg NA, Pritchard JK, Weber JL, Cann HM, Kidd KK, Zhivotovsky LA, Feldman MW (2002). Genetic Structure of Human Populations.

[6] Stoneking M, Delfin F (2010). The human genetic history of East Asia: weaving a complex tapestry. Curr Biol.

[7] Su B, Xiao J, Underhill P, Deka R, Zhang W, Akey J, Huang W, Shen D, Lu D, Luo J, Chu J, Tan J, Shen P, Davis R, Cavalli-Sforza L, Chakraborty R, Xiong M, Du R, Oefner P, Chen Z, Jin L (1999). Y-chromosome evidence for a northward migration of modern humans into eastern Asia during the last ice age. Am J Hum Genet.

[8] Zhang F, Su B, Zhang Y, Jin L (2007). Genetic studies of human diversity in East Asia. Phil Trans R Soc B.

[9] Shi C, Liu Q, Zhao S, Chen H (2019). Ancestry informative SNP panels for discriminating the major east Asian populations: Han Chinese, Japanese and Korean. Ann Hum Genet.

[10] Wang Y, Lu D, Chung Y-J, Xu S (2018). Genetic structure, divergence and admixture of Han Chinese, Japanese and Korean populations. Hereditas.

[11] Japanese Archipelago Human Population Genetics Consortium (2012). The history of human populations in the Japanese archipelago inferred from genome-wide SNP data with a special reference to the Ainu and the Ryukyuan populations. J Hum Genet.

[12] Jinam TA, Kanzawa-Kiriyama H, Saitou N (2015). Human genetic diversity in the Japanese archipelago: dual structure and beyond. Genes Genet Syst.

[13] Watanabe Y, Naka I, Khor S-S, Sawai H, Hitomi Y, Tokunaga K, Ohashi J (2019). Analysis of whole Y-chromosome sequences reveals the Japanese population history in the Jomon period. Sci Rep.

[14] Sato T, Nakagome S, Watanabe C, Yamaguchi K, Kawaguchi A, Koganebuchi K, Haneji K, Yamaguchi T, Hanihara T, Yamamoto K, Ishida H, Mano S, Kimura R, Oota H (2014). Genome-Wide SNP Analysis reveals population structure and demographic history of the Ryukyu islanders in the southern part of the Japanese archipelago. Mol Biol Evol.

[15] Jinam TA, Kanzawa-Kiriyama H, Inoue I, Tokunaga K, Omoto K, Saitou N (2015). Unique characteristics of the Ainu population in northern Japan. J Hum Genet.

[16] Hammer MF, Karafet TM, Park H, Omoto K, Harihara S, Stoneking M, Horai S (2006). Dual origins of the Japanese: common ground for hunter-gatherer and farmer Y chromosomes. J Hum Genet.

[17] Jeong C, Nakagome S, Di Rienzo A (2016). Deep history of east Asian populations revealed through genetic analysis of the Ainu. Genetics.

[18] Kim J, Jeon S, Choi J-P, Blazyte A, Jeon Y, Kim J-I, Ohashi J, Tokunaga K, Sugano S, Fucharoen S, Al-Mulla F, Bhak J (2020). The origin and composition of Korean ethnicity analyzed by ancient and present-day genome sequences. Genome Biology and Evolution.

[19] Kim YJ, Jin HJ (2013). Dissecting the genetic structure of Korean population using genome-wide SNP arrays. Genes Genom.

[20] Jin H-J, Kwak K-D, Hammer MF, Nakahori Y, Shinka T, Lee J-W, Jin F, Jia X, Tyler-Smith C, Kim W (2003). Y-chromosomal DNA haplogroups and their implications for the dual origins of the Koreans. Hum Genet.

[21] Jin H-J, Tyler-Smith C, Kim W (2009). The peopling of Korea revealed by analyses of mitochondrial DNA and Y-chromosomal markers. PLoS One.

[22] Xu S, Yin X, Li S, Jin W, Lou H, Yang L, Gong X, Wang H, Shen Y, Pan X, He Y, Yang Y, Wang Y, Fu W, An Y, Wang J, Tan J, Qian J, Chen X, Zhang X, Sun Y, Zhang X, Wu B, Jin L (2009). Genomic dissection of population substructure of Han Chinese and its implication in association studies. Am J Hum Genet.

[23] Chiang CWK, Mangul S, Robles C, Sankararaman S (2018). A comprehensive map of genetic variation in the World’s largest ethnic group—Han Chinese. Mol Biol Evol.

[24] Wells RS, Yuldasheva N, Ruzibakiev R, Underhill PA, Evseeva I, Blue-Smith J, Jin L, Su B, Pitchappan R, Shanmugalakshmi S, Balakrishnan K, Read M, Pearson NM, Zerjal T, Webster MT, Zholoshvili I, Jamarjashvili E, Gambarov S, Nikbin B, Dostiev A, Aknazarov O, Zalloua P, Tsoy I, Kitaev M, Mirrakhimov M, Chariev A, Bodmer WF (2001). The Eurasian heartland: a continental perspective on Y-chromosome diversity. Proc Natl Acad Sci.

[25] Yao Y-G (2004). Different matrilineal contributions to genetic structure of ethnic groups in the silk road region in China. Mol Biol Evol.

[26] Feng Q, Lu Y, Ni X, Yuan K, Yang Y, Yang X, Liu C, Lou H, Ning Z, Wang Y, Lu D, Zhang C, Zhou Y, Shi M, Tian L, Wang X, Zhang X, Li J, Khan A, Guan Y, Tang K, Wang S, Xu S (2017). Genetic history of Xinjiang’s Uyghurs suggests bronze age multiple-way contacts in Eurasia. Mol Biol Evol.

[27] Lu D, Lou H, Yuan K, Wang X, Wang Y, Zhang C, Lu Y, Yang X, Deng L, Zhou Y, Feng Q, Hu Y, Ding Q, Yang Y, Li S, Jin L, Guan Y, Su B, Kang L, Xu S (2016). Ancestral origins and genetic history of Tibetan highlanders. Am J Hum Genet.

[28] Huerta-Sánchez E, Jin X, Asan BZ, Peter BM, Vinckenbosch N, Liang Y, Yi X, He M, Somel M, Ni P, Wang B, Ou X, Huasang LJ, Cuo ZXP, Li K, Gao G, Yin Y, Wang W, Zhang X, Xu X, Yang H, Li Y, Wang J, Wang J, Nielsen R (2014). Altitude adaptation in Tibetans caused by introgression of Denisovan-like DNA. Nature.

[29] Merriwether DA, Hall WW, Vahlne A, Ferrell RE. mtDNA Variation Indicates Mongolia May Have Been the Source for the Founding Population for the New World. Am J Hum Genet. 1996:9. PMC1915096 8659526

[30] Zerjal T, Xue Y, Bertorelle G, Wells RS, Bao W, Zhu S, Qamar R, Ayub Q, Mohyuddin A, Fu S, Li P, Yuldasheva N, Ruzibakiev R, Xu J, Shu Q, Du R, Yang H, Hurles ME, Robinson E, Gerelsaikhan T, Dashnyam B, Mehdi SQ, Tyler-Smith C (2003). The genetic legacy of the Mongols. Am J Hum Genet.

[31] Qin P, Zhou Y, Lou H, Lu D, Yang X, Wang Y, Jin L, Chung Y-J, Xu S (2015). Quantitating and dating recent gene flow between European and east Asian populations. Sci Rep.

[32] Bai H, Guo X, Narisu N, Lan T, Wu Q, Xing Y, Zhang Y, Bond SR, Pei Z, Zhang Y, Zhang D, Jirimutu J, Zhang D, Yang X, Morigenbatu M, Zhang L, Ding B, Guan B, Cao J, Lu H, Liu Y, Li W, Dang N, Jiang M, Wang S, Xu H, Wang D, Liu C, Luo X, Gao Y, Li X, Wu Z, Yang L, Meng F, Ning X, Hashenqimuge H, Wu K, Wang B, Suyalatu S, Liu Y, Ye C, Wu H, Leppälä K, Li L, Fang L, Chen Y, Xu W, Li T, Liu X, Xu X, Gignoux CR, Yang H, Brody LC, Wang J, Kristiansen K, Burenbatu B, Zhou H, Yin Y (2018). Whole-genome sequencing of 175 Mongolians uncovers population-specific genetic architecture and gene flow throughout North and East Asia. Nat Genet.

[33] Xing J, Wuren T, Simonson TS, Watkins WS, Witherspoon DJ, Wu W, Qin G, Huff CD, Jorde LB, Ge R-L (2013). Genomic analysis of natural selection and phenotypic variation in high-altitude Mongolians. PLoS Genet.

[34] Yang MA, Fan X, Sun B, Chen C, Lang J, Ko Y-C, Tsang C, Chiu H, Wang T, Bao Q, Wu X, Hajdinjak M, Ko AM-S, Ding M, Cao P, Yang R, Liu F, Nickel B, Dai Q, Feng X, Zhang L, Sun C, Ning C, Zeng W, Zhao Y, Zhang M, Gao X, Cui Y, Reich D, Stoneking M, Fu Q. Ancient DNA indicates human population shifts and admixture in northern and southern China. Science. 2020:eaba0909. 10.1126/science.aba0909 32409524

[35] Jeong C, Alkorta-Aranburu G, Basnyat B, Neupane M, Witonsky DB, Pritchard JK, Beall CM, Di Rienzo A (2014). Admixture facilitates genetic adaptations to high altitude in Tibet. Nat Commun.

[36] Zhang C, Lu Y, Feng Q, Wang X, Lou H, Liu J, Ning Z, Yuan K, Wang Y, Zhou Y, Deng L, Liu L, Yang Y, Li S, Ma L, Zhang Z, Jin L, Su B, Kang L, Xu S (2017). Differentiated demographic histories and local adaptations between Sherpas and Tibetans. Genome Biol.

[37] Deng L, Xu S (2018). Adaptation of human skin color in various populations. Hereditas.

[38] Peng Y, Shi H, Qi X, Xiao C, Zhong H, Ma RZ, Su B (2010). The ADH1B Arg47His polymorphism in east Asian populations and expansion of rice domestication in history. BMC Evol Biol.

[39] Takeuchi F, Katsuya T, Kimura R, Nabika T, Isomura M, Ohkubo T, Tabara Y, Yamamoto K, Yokota M, Liu X, Saw W-Y, Mamatyusupu D, Yang W, Xu S, Teo Y-Y, Kato N, Japanese genome variation consortium (2017). The fine-scale genetic structure and evolution of the Japanese population. PLoS ONE.

[40] Hlusko LJ, Carlson JP, Chaplin G, Elias SA, Hoffecker JF, Huffman M, Jablonski NG, Monson TA, O’Rourke DH, Pilloud MA, Scott GR (2018). Environmental selection during the last ice age on the mother-to-infant transmission of vitamin D and fatty acids through breast milk. Proc Natl Acad Sci U S A.

[41] Simonson TS, Yang Y, Huff CD, Yun H, Qin G, Witherspoon DJ, Bai Z, Lorenzo FR, Xing J, Jorde LB, Prchal JT, Ge R (2010). Genetic evidence for high-altitude adaptation in Tibet. Science.

[42] Project TMMJRP, Nagasaki M, Yasuda J, Katsuoka F, Nariai N, Kojima K, Kawai Y, Yamaguchi-Kabata Y, Yokozawa J, Danjoh I, Saito S, Sato Y, Mimori T, Tsuda K, Saito R, Pan X, Nishikawa S, Ito S, Kuroki Y, Tanabe O, Fuse N, Kuriyama S, Kiyomoto H, Hozawa A, Minegishi N, Douglas Engel J, Kinoshita K, Kure S, Yaegashi N, Yamamoto M (2015). Rare variant discovery by deep whole-genome sequencing of 1,070 Japanese individuals. Nat Commun.

[43] Moon S, Kim YJ, Han S, Hwang MY, Shin DM, Park MY, Lu Y, Yoon K, Jang H-M, Kim YK, Park T-J, Song DS, Park JK, Lee J-E, Kim B-J (2019). The Korea biobank Array: design and identification of coding variants associated with blood biochemical traits. Sci Rep.

[44] Jeon S, Bhak Y, Choi Y, Jeon Y, Kim S, Jang J, Jang J, Blazyte A, Kim C, Kim Y, Shim J, Kim N, Kim YJ, Park SG, Kim J, Cho YS, Park Y, Kim H-M, Kim B-C, Park N-H, Shin E-S, Kim BC, Bolser D, Manica A, Edwards JS, Church G, Lee S, Bhak J (2020). Korean Genome Project: 1094 Korean personal genomes with clinical information. Sci Adv.

[45] Xue F, Wang Y, Xu S, Zhang F, Wen B, Wu X, Lu M, Deka R, Qian J, Jin L (2008). A spatial analysis of genetic structure of human populations in China reveals distinct difference between maternal and paternal lineages. Eur J Hum Genet.

[46] Gao Y, Zhang C, Yuan L, Ling Y, Wang X, Liu C, Pan Y, Zhang X, Ma X, Wang Y, Lu Y, Yuan K, Ye W, Qian J, Chang H, Cao R, Yang X, Ma L, Ju Y, Dai L, Tang Y (2020). The Han100K initiative, Zhang G, Xu S. PGG.Han: the Han Chinese genome database and analysis platform. Nucleic Acids Res.

[47] Cao Y, Li L, Xu M, Feng Z, Sun X, Lu J, Xu Y, Du P, Wang T, Hu R, Ye Z, Shi L, Tang X, Yan L, Gao Z, Chen G, Zhang Y, Chen L, Ning G, Bi Y, Wang W, The ChinaMAP Consortium (2020). The ChinaMAP analytics of deep whole genome sequences in 10,588 individuals. Cell Res.

[48] Zhang C, Gao Y, Liu J, Xue Z, Lu Y, Deng L, Tian L, Feng Q, Xu S (2018). PGG.Population: a database for understanding the genomic diversity and genetic ancestry of human populations. Nucleic Acids Res.

[49] Xu S, Li S, Yang Y, Tan J, Lou H, Jin W, Yang L, Pan X, Wang J, Shen Y, Wu B, Wang H, Jin L (2011). A genome-wide search for signals of high-altitude adaptation in Tibetans. Mol Biol Evol.

[50] Lou H, Lu Y, Lu D, Fu R, Wang X, Feng Q, Wu S, Yang Y, Li S, Kang L, Guan Y, Hoh B-P, Chung Y-J, Jin L, Su B, Xu S. A 3.4-kb Copy-Number Deletion near EPAS1 Is Significantly Enriched in High-Altitude Tibetans but Absent from the Denisovan Sequence. Am J Hum Genet 2015;97:54–66. 10.1016/j.ajhg.2015.05.005 PMC4572470 26073780

[51] Ouzhuluobu HY, Lou H, Cui C, Deng L, Gao Y, Zheng W, Guo Y, Wang X, Ning Z, Li J, Li B, Bai C, Baimakangzhuo G, Dejiquzong B, Duojizhuoma LS, Wu T, Xu S, Qi X, Su B (2020). De novo assembly of a Tibetan genome and identification of novel structural variants associated with high-altitude adaptation. Natl Sci Rev.

[52] Barrett R, Schluter D (2008). Adaptation from standing genetic variation. Trends Ecol Evol.

[53] McCoy RC, Akey JM (2017). Selection plays the hand it was dealt: evidence that human adaptation commonly targets standing genetic variation. Genome Biol.

[54] Schrider DR, Kern AD (2017). Soft sweeps are the dominant mode of adaptation in the human genome. Mol Biol Evol.

[55] Akiyama M, Ishigaki K, Sakaue S, Momozawa Y, Horikoshi M, Hirata M, Matsuda K, Ikegawa S, Takahashi A, Kanai M, Suzuki S, Matsui D, Naito M, Yamaji T, Iwasaki M, Sawada N, Tanno K, Sasaki M, Hozawa A, Minegishi N, Wakai K, Tsugane S, Shimizu A, Yamamoto M, Okada Y, Murakami Y, Kubo M, Kamatani Y (2019). Characterizing rare and low-frequency height-associated variants in the Japanese population. Nat Commun.

[56] Mérot C, Oomen RA, Tigano A, Wellenreuther M (2020). A roadmap for understanding the evolutionary significance of structural genomic variation. Trends Ecol Evol.

[57] Lee S, Seo J, Park J, Nam J-Y, Choi A, Ignatius JS, Bjornson RD, Chae J-H, Jang I-J, Lee S, Park W-Y, Baek D, Choi M (2017). Korean variant archive (KOVA): a reference database of genetic variations in the Korean population. Sci Rep.

[58] Manolio TA, Collins FS, Cox NJ, Goldstein DB, Hindorff LA, Hunter DJ, McCarthy MI, Ramos EM, Cardon LR, Chakravarti A, Cho JH, Guttmacher AE, Kong A, Kruglyak L, Mardis E, Rotimi CN, Slatkin M, Valle D, Whittemore AS, Boehnke M, Clark AG, Eichler EE, Gibson G, Haines JL, Mackay TFC, McCarroll SA, Visscher PM (2009). Finding the missing heritability of complex diseases. Nature.

